# Five-year clinical outcomes of RefluxStop surgery in the treatment of acid reflux: a prospective multicenter trial of safety and effectiveness

**DOI:** 10.1007/s00464-025-11979-9

**Published:** 2025-07-22

**Authors:** László Harsányi, Zsolt Kincses, Milan Veselinović, Joerg Zehetner, Áron Altorjay

**Affiliations:** 1 Department of Surgery, Transplantation and Gastroenterology, Semmelweis University, Budapest, Hungary; 2 The Department of Surgery Kenezy Campus, Clinical Center of the University of Debrecen Teaching Hospital, Debrecen, Hungary; 3 Department of Minimally Invasive Upper Digestive Surgery, University Hospital for Digestive Surgery–First Surgical Hospital, Clinical Center of Serbia, Belgrade, Serbia; 4 Department of Visceral Surgery, Hirslanden Clinic Beau-Site, Bern, Switzerland; 5 Surgical Department, Fejér County Szent György University Teaching Hospital, Székesfehérvár, Hungary

**Keywords:** Gastroesophageal reflux disease, Antireflux surgery, Quality of life, Proton pump inhibitors, 24-h pH monitoring, Dysphagia

## Abstract

**Introduction:**

RefluxStop surgery corrects all three components of the anti-reflux barrier without affecting the food passageway. Long-term 5-year safety and effectiveness clinical outcomes are presented from the RefluxStop CE mark trial, used in an FDA PMA submission. This comprehensive and meticulously controlled data are, therefore, presented across multiple reports with other outcomes in a separate complementary article.

**Methods:**

A prospective, single-arm, multicenter clinical study was conducted to investigate RefluxStop surgery in 50 adults with chronic GERD, PPI use, GERD-HRQL score, 24-h pH testing, contrast-swallow x-ray, and serious/non-serious AEs presented.

**Results:**

Forty-four (*n* = 44) subjects completed 5-year follow-up, whereof 91% underwent pH testing and contrast-swallow x-ray. Three subjects were withdrawn due to COVID-19 (two deaths and one long-COVID), all well-treated beforehand, at 3–4 years. PPI usage (including COVID-19 subjects) in 1/47 (2.1%). The median (IQR) total GERD-HRQL score improved by 90% (72–98%) from a baseline of 29.5 (33.0–24.0) to 3.0 (0.5–7.5) at 5 years (*p* < 0.001) and 24-h pH monitoring results improved by 90.4% to a mean total acid exposure time (pH < 4) of 1.57% from 16.35% at baseline (*p* < 0.001). No cases (0%) of device explantation, migration/erosion, or esophageal dilatation occurred during the study. Five-year contrast-swallow x-ray showed zero (0%) dislocations, migrations, or re-herniations. Five subjects (*n* = 5) experienced serious AEs of which all were resolved. Only two procedure-related AEs occurred between 1 and 5 years, one moderate dyspepsia and one mild dysphagia, subsequently resolved.

**Conclusion:**

The RefluxStop procedure demonstrated exceptional long-term 5-year outcomes. Only one subject took PPIs at follow-up. Both median GERD-HRQL scores and mean 24-h pH results improved by > 90% from baseline (*p* = 0.001). No device-related AEs, explantations, or migrations occurred during the 5-year study. Only two procedure-related AEs occurred between 1 and 5 years, one dyspepsia and one mild dysphagia (resolved). These objective and patient-reported results were robustly maintained from previously published 1- to 4-year data.

Gastroesophageal reflux disease (GERD) is an inherently chronic condition that often necessitates lifelong medical therapy with proton pump inhibitors (PPIs), however, such therapy is plagued by serious consequences for many sufferers. The literature includes many articles regarding the serious adverse effects and insufficient therapeutic effect of long-term PPI use. One such analysis that summarizes the side effect profile of PPIs by following over 157,000 United States (US) veterans for 10 years of PPI use was published by Xie and colleagues in 2019 [1]. This large and long-term study showed that more than 7,000 extra deaths occurred by adverse effects of PPI use. The four primary causes of death associated with PPI use were, in descending order: (1)* cardiovascular events* via drug-related endothelial aging [2], supported by systematic literature review of 37 separate studies [3]; (2)* digestive tract malignancy*, where a database analysis reported all deaths caused by esophageal cancer in Sweden with 38% of which preceded by PPI use [4], as well as showing a strong correlation between gastric cancer and PPI use [5]; (3)* renal disease*, where PPI medications are nephrotoxic, impairing kidney function [6]; and (4) *infectious or parasitic disease*, where the protective barrier provided by gastric acid is lacking due to the underlying function of PPI medications [7]. Aside from the plenitude of other adverse outcomes associated with PPIs, therapy fails to treat symptoms in about 40% of cases [8], both subjectively where > 50% complain of intermittent heartburn [9] and objectively where as many as 39% of PPI users may fail 24-h pH testing while receiving PPI therapy [9].

In the world today, about 1 billion persons suffer from acid reflux [10]. Despite this enormous figure, as well as the serious side effects and considerable failure rate of PPI therapy, surgical treatment of GERD has not flourished and less than 1% of patients are offered anti-reflux surgery [11]. The reason for this is logical given that current surgical treatment of GERD has side effects that often lead to patient dissatisfaction after operation. The standard-of-care anti-reflux procedure, Nissen fundoplication, and other conventional surgical techniques, like magnetic sphincter augmentation (MSA), are associated with postoperative adverse events (AEs) since they involve wrapping of the fundus around the distal esophagus or augmentation of the lower esophageal sphincter (LES) via magnetic ring, respectively. This mechanism of action may increase the risk of postoperative dysphagia or the inability to belch/vomit [12] with an extensive literature review of Nissen fundoplication presenting a dysphagia rate of 29% after 5 years [13], and conventional techniques are associated not only with unwanted sequelae but also diminished efficacy over time [8, 14–16]. 

The mechanisms normally preventing acid reflux have not been sufficiently understood in the past. A leap forward was taken by way of two white papers published by the American Foregut Society [17, 18] in recent years, which outline the three main contributors to the anti-reflux barrier (ARB): the crural diaphragm, LES and its sling fibers, and the gastroesophageal flap valve created by the acute angle of His. The RefluxStop technique, an emerging medical technology and procedure, employs a pioneering mechanism of action that comprehensively restores all three attributes of the ARB (Fig. 1) without esophageal encirclement or compression, the a priori rationale for low rates of unwanted sequelae like dysphagia and gas-bloating. Clinical experience with the RefluxStop procedure suggests that one parameter of the ARB may have more importance than others, namely LES position. The constant and repetitive pressure variations in the chest cavity of respiration permeate the hiatal opening and affect the LES, particularly when positioned close to the diaphragmatic hiatus.

**Fig. 1:**
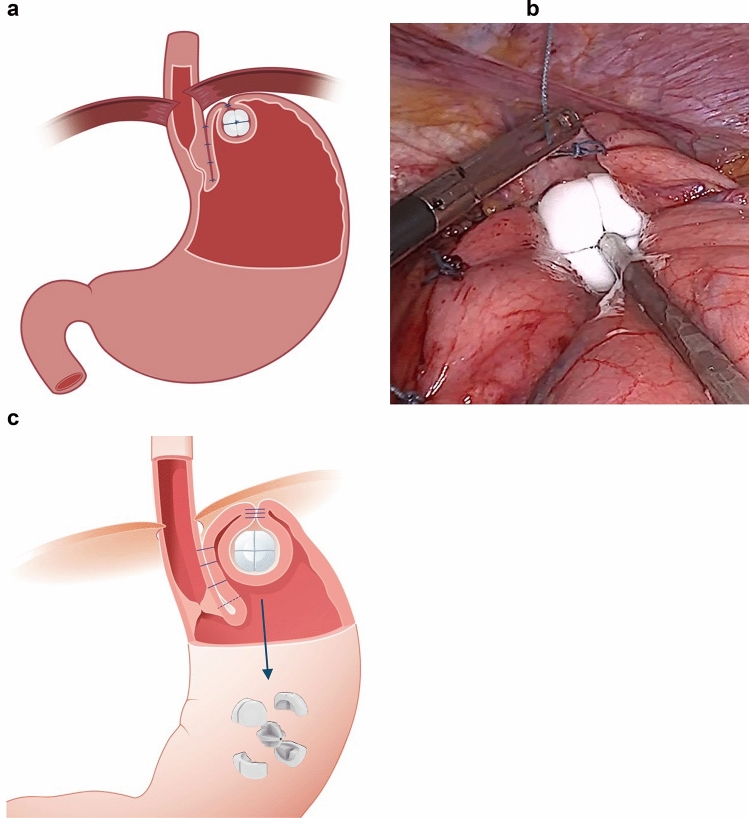
**a** Anatomic representation of the results of RefluxStop surgery. Following extensive mediastinal and fundic dissection, the lower esophageal sphincter (LES) is mobilized intraabdominally, cruroplasty and narrow esophagogastroplication (90–120°) are performed, and the device is fully invaginated in an exterior fundic pouch. This concert of steps comprehensively reconstructs the ARB without encircling the lower esophagus. **b** Intraoperative image of the RefluxStop device placed by its dedicated deployment tool in the fundic invagination pouch to be loosely closed with suturing. **c** In the unlikely event of migration/penetration (although no such case occurred in this study) that may occur due to overly tight suturing of the invagination pouch, the device is designed to asymptomatically pass through the digestive tract (as shown by a couple of cases from independent real-world data)

The short-term (i.e., 1-year) results of the RefluxStop CE mark trial published by Bjelović, et al. [19] and mid- to long-term (i.e., 3- and 4-year) results of the trial have been published by Harsanyi et al. [20, 21], showing favorable clinical outcomes with clear effectiveness and a low-risk safety profile sustained over 4 years. Besides the previously published 1-, 3-, and 4-year data from the RefluxStop trial [19–21], several real-world studies from Germany, Switzerland, the United Kingdom, and Austria have shown consistent safety and effectiveness after RefluxStop surgery despite substantial variability in patient demographics. Investigation with larger samples and longer follow-up had shown contextually remarkable results in those with comorbid subgroup populations, such as those with variable hiatal hernia sizes (4–10 cm) [22] and/or ineffective esophageal motility (IEM) [23] with mid-term (i.e., 3–4 years) follow-up. The purpose of this report is to present the first long-term (i.e., mean 5.7 years) outcomes of RefluxStop surgery from its CE mark trial.

## Methods

### Study design and objectives

This was a prospective, single-arm, multicenter clinical study with trial registration number NCT02759094 (https://clinicaltrials.gov/study/NCT02759094). The design of the study has previously been reported in detail [19]. A total of 50 subjects with chronic GERD were included in the study, having symptoms for at least 6 months, confirmed by 24-h pH monitoring. Patients with hiatal hernia > 3 cm were excluded from the study. The operation was performed between December 2016 and September 2017 at four centers. Further details regarding earlier follow-up results have been published [19–21]. In general, data are presented both as Per Protocol (PP) and as Full Analysis Set (FAS). The RefluxStop procedure was performed as described in the publication of 1-year follow-up outcomes [19]. Specific attributes and considerations of RefluxStop surgery are discussed in this article as several learning points have been elucidated by experience in clinical practice. The study included the learning curve effect for both a new procedure and for individual surgeons.

Through laparoscopic access, RefluxStop surgery includes repositioning of hiatal hernia (if present), extensive mediastinal dissection of the esophagus with a vagal- and pleura-conserving approach (to attain at least 5 cm of intraabdominal length), hiatal hernia repair with cruroplasty, dissection of the gastric fundus with division of four fundic short gastric vessels, and posterior dissection of the fundus to achieve a tension-free and ‘floppy’ fundus. Plication of the fundus and esophagus was executed between the vagal trunks on the patient’s left side, on 90–120° of the esophageal circumference, thus not encircling the esophagus. Suturing > 140° was contraindicated in this study. The RefluxStop device was then loosely and fully invaginated in a pouch on the outside of the gastric fundus at its most superior aspect, and close to esophagus. It is paramount to avoid overly tight suturing of the fundic pouch around the device to prevent potential device migration/erosion into the stomach cavity. The position of the device should be in proximity to the esophagus and with its entire body above the upper edge of the LES (Fig. 1). As such, the upper edge of the device should be positioned at least 4 cm above the angle of His. Dysphagia may be particularly avoided by the RefluxStop procedure since it does not encircle or put pressure on the esophagus, as long as crural repair is not performed too tightly, and instead a looser hiatus closure than in Nissen fundoplication should be performed. The supplied deployment tool allows the surgeon to position and maintain the RefluxStop device in place during its invagination.

AEs were recorded at annual (i.e., years 1–5) and non-scheduled supplementary visits. Patient-reported outcomes were assessed annually via a battery of questions from the GERD Health-Related Quality of Life (GERD-HRQL) questionnaire providing a total score between 0 and 50, as well as questions related to other parameters such as regurgitation, inability to belch and/or vomit, and PPI use. At the 5-year visit, further evaluation was conducted via 24-h pH monitoring with acid exposure time and contrast-swallow x-ray imaging to assess for re-herniation, migration, or dislocation of the device. If therapy failure occurred, defined as PPI use or < 50% improvement of GERD-HRQL score since baseline, additional 24-h pH monitoring and contrast-swallow x-ray were performed. Additionally, gastroscopy was performed if pH testing was normal, and manometry was performed per the surgeon’s assessment/discretion. All secondary endpoints were included in the 5-year follow-up.

### Statistical analysis

Since this study has been used both for receiving CE mark in Europe and its Food and Drug Administration (FDA) Premarket Approval (PMA) submission in the United States (US) using 5-year outcomes, the quantity and quality of data collected are extensive in addition to being rigorously controlled with a high rate of follow-up. Data analysis was much expanded and includes validation of important steps. All data were audited by two independent third parties including a US-based FDA expert consultancy firm that compared patient journals with the electronic case report form (eCRF) platform, Viedoc, which was programmed and maintained by another third party, Link Medical AB. The deviations log was further thoroughly analyzed by both the independent Contract Research Organization (CRO) and Link Medical. All tables and figures were generated according to the Statistical Analysis Plan (SAP) by Link Medical. All data were programmed by a third party in cooperation with Link Medical and transferred to tables and figures directly from the data system without manual handling. This data transfer process was extensively validated. Statistical analysis was conducted on the following data sets: Intention to treat, Full Analysis Set (FAS)—all subjects that underwent device implantation (*n* = 50), based on modified intention-to-treat (mITT); and Per Protocol (PP) analysis set—all subjects at follow-up.

For the primary endpoints, the total GERD-HRQL score and 95%-confidence intervals (CIs) for the percentage of patients with ≥ 50% improvement in total GERD-HRQL score, were calculated via the Clopper-Pearson exact method formula. To test for significant differences between data of multiple follow-ups, change-from-baseline data were first tested for equality of variances via the Levene’s test. In case of equal variances, differences from baseline were assessed via paired t-test and, in addition, repeated measures analysis of variance (ANOVA); otherwise, via the Wilcoxon signed rank test. To address multiplicity (i.e., due to testing several time points for total GERD-HRQL score), additional analyses adjusted via the Benjamini–Hochberg procedure were generated and it was confirmed that no adjusted *p*-value shifted the conclusion from statistically significant to non-significant compared to unadjusted *p*-values. The significance level was 0.05.

For the secondary effectiveness endpoint of acid exposure time measured by 24-h pH monitoring, the percentage of overall time with pH < 4 was analyzed by Wilcoxon signed rank test comparing baseline and follow-up visits.

### Ethical approval

The study was conducted in accordance with the Declaration of Helsinki and the Regional Ethics Committees approved the study protocol: the Medical Research Council (MRC), Scientific and Research Ethics Committee (SREC), Budapest, Hungary; and the Ethics Committee of Serbia (EOS), Belgrade, Serbia. All subjects provided written informed consent for study participation that included publication of anonymized data.

## Results

Table 1 summarizes key effectiveness and safety results of the study at 5 years, unless otherwise stipulated.

**Table 1:** Summary of effectiveness and safety outcomes at 5 years, unless otherwise specified

Total GERD-HRQL score:	90% (72–98%) median (IQR) improvement from a baseline of 29.5 (24.0–33.0) to 3.0 (0.5–7.5) at 5-year follow-up (*p* < 0.001)
pH monitoring:	90.4% improvement in mean total time with pH < 4 from 16.5% at baseline to 1.57% at 5 years (*p* < 0.001)
Regular daily PPI usage:	97.9% (*n* = 46/47) of subjects were not using PPIs at 5 years, with inclusion of 3- and 4-year data carried forward for three (*n* = 3) COVID-19 subjects at 5-year follow-up (i.e., two deaths and one bedbound with long-COVID)
Contrast-swallow imaging:	At 5-year x-ray: No cases of re-herniation, dislocation, or migration/erosion occurred
Device explant, migration, or esophageal dilatations: Severe SAEs:	No device explant, migration/erosion, or esophageal dilatation occurred during the entire study Two severe SAEs occurred, one subject with bleeding from short gastric vessels and one subject with an infection with abscesses not spread to the fully encapsulated implant, both satisfactorily resolved in fully treated subjects
Dysphagia and other food passageway-related AEs:	Food passageway-related AEs (i.e., odynophagia, odynophagia, inability to belch/vomit and gas-bloating) are presented in a separate article [26] due to the magnitude of data and analysis used for both CE mark and FDA PMA submission of 5-year data AE dysphagia, between 2 weeks of surgical recovery and the 5-year study conclusion, occurred in one (2.1%) subject with a mild dysphagia case that resolved before the 5-year visit

*AE* adverse event; *GERD-HRQL* Gastroesophageal Reflux Disease Health-Related Quality of Life; *IQR* interquartile range; *PPI* proton pump inhibitor; *SAE* serious adverse event

### Patient population

The RefluxStop procedure was performed on 50 subjects. A total of 44 subjects completed 5-year follow-up (i.e., mean 5.7 years) and were included in the PP and FAS analyses. At baseline, the mean (SD) age was 51.5 (11.8) years, the mean (SD) weight was 78.2 (14.7) kg, the mean (SD) body mass index (BMI) was 26.81 (4.41) kg/m^2^, 56% were male, and the mean (SD) hiatal hernia size was 2.51 (0.58) cm.

### Missing subjects

Follow-up data were available for 44/50 subjects at 5-year follow-up. A summary of the missing patients is provided below:

- Two subjects (*n* = 2) died from COVID-19 and one (*n* = 1) missed 5-year follow-up due to being predominantly bedbound with long-COVID. All three COVID-19 subjects were well-treated before COVID-19 at 3- (n = 2) and 4-year (*n* = 1) follow-up: all three were satisfied with treatment, had substantially lower GERD-HRQL scores with an 88–95% range of improvement, did not use PPIs, and had regurgitation classification of *None*. Thus, all parameters indicated that these three subjects were well-treated.
- Three (*n* = 3) subjects terminated the study in the first year (i.e., one at 3 months and two at 6 months):
  o Two (*n* = 2) subjects were satisfied, did not use PPIs, had a low average GERD-HRQL score of average 2.5, and did not experience any regurgitation.
  o One (*n* = 1) subject ended the study dissatisfied with a high GERD-HRQL score, albeit without using regular daily PPI therapy at the 6-month follow-up or having any regurgitation at 6 months. This subject experienced a broken needle that was left subcutaneously, which was surgically removed, and refused 24-h pH monitoring, which did not clarify if treatment failure occurred.

Of the remaining 44 subjects that completed follow-up with the GERD-HRQL questionnaire and AE reporting, 40 (91%) also completed 24-h pH testing and contrast-swallow x-ray imaging. Of the four subjects that completed the GERD-HRQL questionnaire but did not perform the physical tests, one subject abstained due to severe illness (unrelated to acid reflux), one subject was outside of the country, one subject refused, and one could not be reached at the time of testing. The clinical status of these four subjects is described below:

- Three (*n* = 3) of the subjects that completed follow-up without 24-h pH testing were satisfied, did not utilize PPI therapy, did not experience regurgitation, and had significant improvements in GERD-HRQL scores ranging from 83 to 94%.
- One (*n* = 1) subject refused to continue due to severe illness (unrelated to acid reflux surgery). At the 4-year follow-up (before the subject fell ill), the subject did not use PPI therapy, did not experience regurgitation, and had ≥ 50% improvement in GERD-HRQL score as well as earlier pH results at 6 months (i.e., acid exposure time of 1.1%, which improved from 10.6% at baseline). After the onset of the other illness, the subject experienced moderate regurgitation and utilized PPI therapy at the 5-year follow-up. This is the only subject in the study that took PPIs at 5 years, however, lack of 24-h pH monitoring precludes clarity of the reason.

## Effectiveness outcomes

### Primary effectiveness outcome: reduction in total GERD-HRQL score

**Fig. 2:**
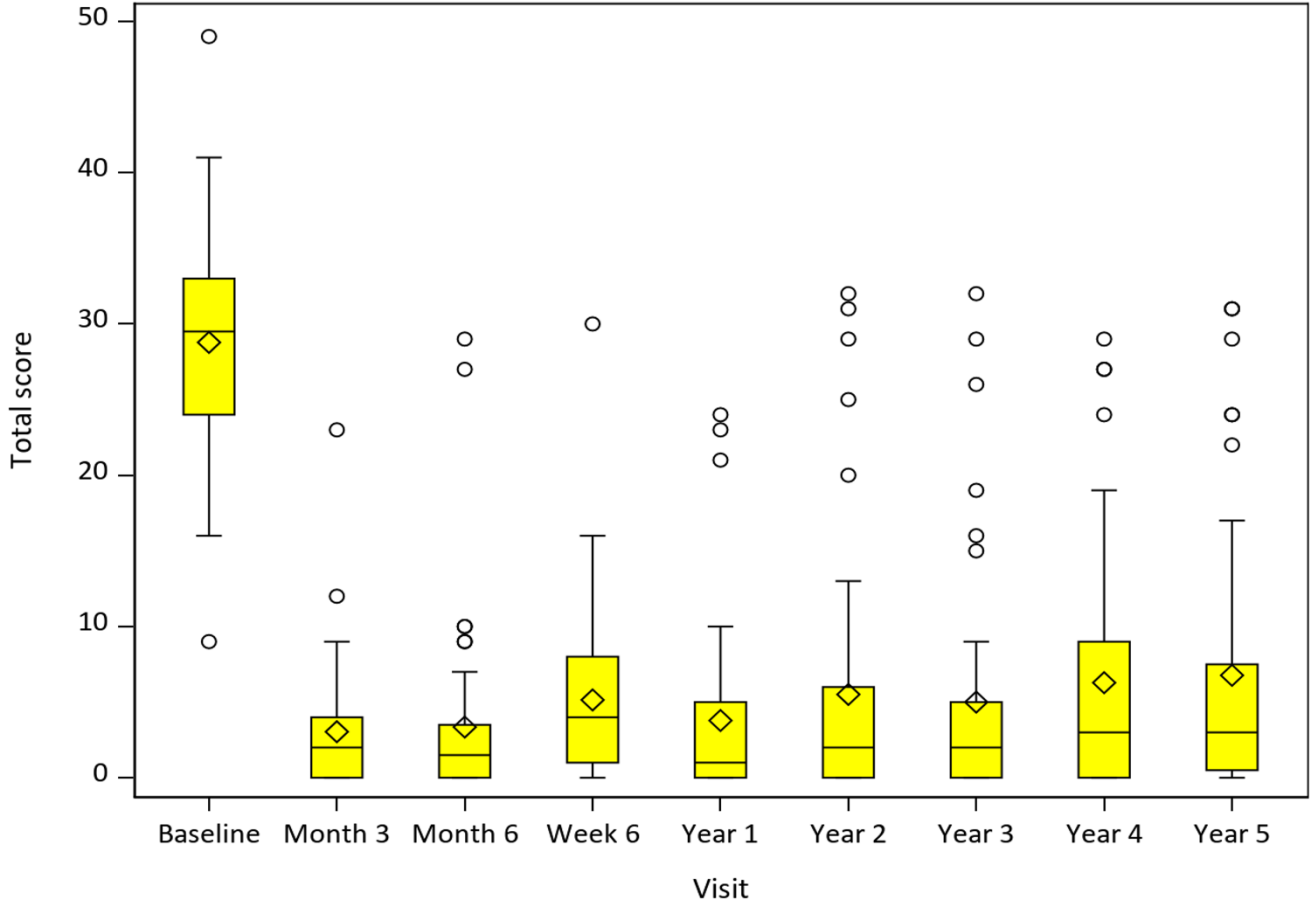
Total GERD-HRQL scores between baseline and 5-year follow-up. The median scores were substantially improved from baseline and remained consistent over time. The outlier values were in general not related to acid reflux as verified by 24-h pH monitoring (most common reason for higher GERD-HRQL scores being gastritis experienced by 8 patients). Only one of the subjects with < 50% improvement of GERD-HRQL score had a pathologic total time of 24-h pH monitoring. Percentage improvements for each annual visit are provided below: **1 year (*n* = 42):** median 95.1% (83.8–100%) improvement in total GERD-HRQL score; follow-up median score of 1.0 (0–5) from baseline score of 29.5. **2 years (*n* = 47):** median 93.1% (78.6–100%) improvement in total GERD-HRQL score; follow-up median score of 2.0 (0–6) from baseline score of 29.5. **3 years (*n* = 47):** median 93.1% (78.3–100%) improvement in total GERD-HRQL score; follow-up median score of 2.0 (0–5) from baseline score of 29.5. **4 years (*n* = 46):** median 90.3% (69–100%) improvement in total GERD-HRQL score; follow-up median score of 3.0 (0–9) from baseline score of 29.5. **5 years (*n* = 44):** median 90% (72–98%) improvement in total GERD-HRQL score; follow-up median score of 3.0 (0.5–7.5) from baseline score of 29.5. Main reason for higher GERD-HRQL scores is gastritis diagnosed in 8 patients. The central line within each box indicates the median score, while the diamond symbol represents the mean score. The boundaries of each box correspond to the 25th (bottom) and 75th (top) percentiles, and the whiskers extend to the range of nonoutlier values. Circles beyond the whiskers denote outlier values. Only one of these subjects had a pathologic total time of 24-h pH monitoring

The median (IQR) improvement in total GERD-HRQL score (*n* = 44) of 90% (72–98%) was based on a baseline score of 29.5 (24.0–33.0) reduced to 3.0 (0.5–7.5) at 5-year follow-up (*p* < 0.001), as depicted in Fig. 2. As illustrated, the GERD-HRQL scores significantly decreased shortly after surgery and were sustained in the long-term for up to 5 years. Only one subject had < 50% improvement in total GERD-HRQL score and pathologic 24-h pH total time with pH < 4 at 5 years (Table 2). When including the latest follow-up results at 3–4 years of the three subjects lost to follow-up due to COVID-19, the mean total GERD-HRQL score is reduced by 0.03 more. A primary reason for higher total GERD-HRQL scores is gastritis, a diagnosis made in 8 of the 12 subjects in the study with gastritis symptoms (Table 3).

**Table 2:** GERD-HRQL treatment success* with failure cases validated by 24-h pH testing**–Full analysis set

Time (months)	Group	Subjects with end of study treatment success	Study Success definition	*n* subjects	Comment	*p*-value
End of study	FAS	**97.9%**	Successful GERD-HRQL subjects plus those with < 50% improved GERD-HRQL score with normal 24-h pH testing	**47/50**	**End of study follow-up** (5-yr *n* = 44, 4-year *n* = 2, and 3-yr *n* = 1)***	< 0.001

*FAS *full analysis set

^*^Not successful result defined as total GERD-HRQL score < 50% improved from baseline

^**^If patient-reported GERD-HRQL score < 50% improved from baseline and objective 24-h pH is normal, subject is regarded as treatment success. One reason for such an outcome is gastritis, which was diagnosed in eight subjects in this study

^***^Three well-treated subjects lost to COVID-19, two deaths and one bedbound with long-COVID, with 3–4-year data before COVID-19 used

Bold text highlights key results described in the Results section

**Table 3:** Subjects with gastritis–full analysis set

Subjects with gastritis out of the total (*N* = 50)	*n* (%)	*m*
Any gastritis diagnosis plus subjects with gastritis-symptom AE epigastric pain	12 (24.0%)	13
Any gastritis diagnosis three-quarter based on endoscopy	8 (16.0%)	9
Any gastritis by severity		
Mild	5 (10.0%)	6
Moderate	3 (6.0%)	3
Severe	0 (0%)	0
Additional subjects with main symptom of gastritis: AE epigastric pain	4 (8%)	4

*AE* adverse event

### Secondary effectiveness outcomes

#### Objective 24-h pH monitoring

**Fig. 3:**
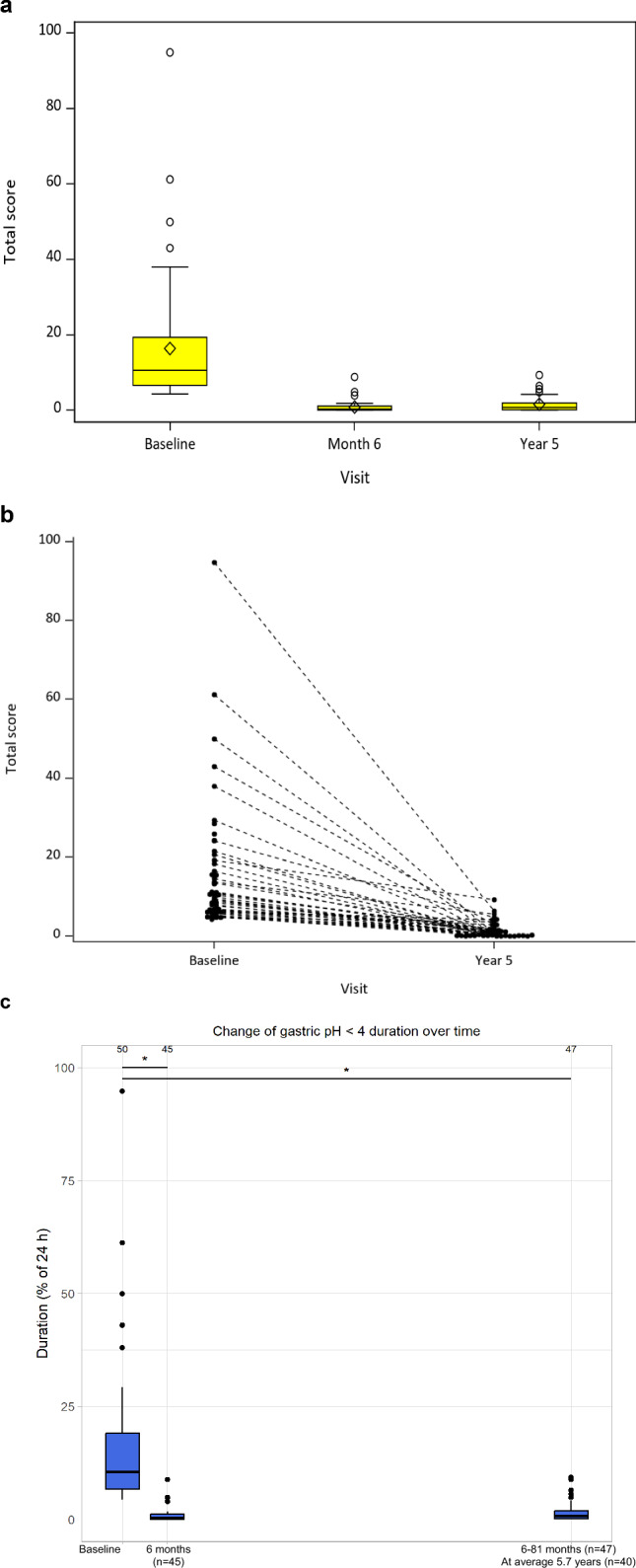
**a** Acid exposure time on 24-h pH monitoring comparing baseline, 6-month, and 5-year follow-up values (*p* < 0.001). **b** 24-h pH monitoring results comparing individual subjects’ (with one dashed line each) providing acid exposure at baseline and 5 years (*p* < 0.001). Mean total time pH < 4 was reduced from 16.35% at baseline to 1.57% at year 5. As one could see from the Fig. 3a below the 5-year results is very stable comparing 5-years and 6 months results. **c** Esophageal pH < 4 (*n* = 47/50) duration over time ranging between 6 and 81 months using Last Observation Carried Forward (LOCF) methodology up to 5 years, of which *n* = 40 subjects had a mean of follow-up of 5.7 years (p < 0.001). The central line within the box indicates the median score, while the diamond symbol represents the mean score. The boundaries of the box correspond to the 25th (bottom) and 75th (top) percentiles, and the whiskers extend to the range of nonoutlier values. Circles beyond the whiskers denote outlier values. Each dashed line represents an individual subject’s acid exposure at baseline and 5 years. Using normal pH as defined in the LINX FDA Memorandum (total time pH < 4 of ≥ 4.5% or ≥ 50% reduction of total time) [24] in this study provides 100% normal pH outcome at year 5. Applying normal value of total time of 5.9% when pH < 4, two subjects failed this pH test. (Research has shown that wireless Bravo capsules have a higher sensitivity for pH measurement than via nasogastric tube, a logical result presenting the normal value to be 5.9% of the 95% level of values in the normal population [25]). Note: For the 47 subjects that underwent pH testing at any time point after surgery, a Last Observation Carried Forward (LOCF) approach was applied, imputing the pH measurement for the 5-year assessment. The central line within each box indicates the median duration. The boundaries of each box correspond to the 25th (bottom) and 75th (top) percentiles, and the whiskers extend to the range of non-outlier values. Circles beyond the whiskers denote outlier values. This graph includes all 47 subjects that underwent pH testing any time after surgery (LOCF up to 5 years). Forty out of 47 subjects have mean 5.7 years follow-up. Numbers on top indicate subject numbers at each time point

The lower esophageal acid exposure time in all subjects is presented in Fig. 3a. A significant reduction in the duration of exposure was observed at 6 months (*p* < 0.001) and 5 years (*p* < 0.001) compared to baseline. Specifically, the mean (SD) duration decreased from 16.35% (16.60%) at baseline to 1.57% (2.10%) at 5 years. This represents a 90.4% reduction at 5 years. For additional context, the mean duration of 1.57% at 5 years is substantially lower than the pathologic threshold value. Figure 3b demonstrates the lower esophageal acid exposure time represented as the percentage of time with pH < 4 over a 24-h period, shown with one line per patient and measured at baseline and 5 years postoperatively. The downward trendlines demonstrate that all subjects had a significant reduction in acid exposure by 5 years. Figure 3c, which includes all subjects that at any time after surgery performed 24-h pH monitoring (*n* = 47), exhibits nearly identical results to the analysis of data from exclusively 5-year follow-up, presented in Fig. 3a.

With application of the parameters for normal 24-h pH outcomes that were used in the FDA Executive Summary Memorandum for the MSA device [24], defined as total time with pH < 4 of < 4.5% *or* ≥ 50% improvement of the total time with pH < 4 from baseline, based on this definition, all subjects in the present study had normal pH values at 5 years. By instead using the research definition posited by Pandolfino et al. (2003) [25] for normal values during 24-h Bravo wireless capsule-measurement (i.e., used in this study) of ≤ 5.9%, two (*n* = 2) subjects failed 24-h pH testing. Bravo capsules are more sensitive than conventional nasogastric catheter solutions (as assessed in healthy individuals with 95th percentile of total time pH < 4 being higher than the normal threshold of 4.5%). Finally, if the standard 4.5% level for nasogastric tubes (not used in this study) is utilized for analysis, normalization of pH testing occurred in all but four (*n* = 4) subjects at 5 years. However, three out of four (*n* = 3/4) such subjects in this study were considered exceptionally well-treated at 5 years when considering all other parameters. See a clinical summary for these subjects below:

- **Subject 1:** Satisfied; PPI Never; Regurgitation, None; GERD-HRQL score baseline/5 years = 48/1 (98% improvement); total time pH < 4 was 5.6%, pH improvement from baseline of 59.4%.
- **Subject 2:** Satisfied; PPI Never; Regurgitation, None; GERD-HRQL score baseline/5 years = 37/6 (84% improvement); total time pH < 4 was 4.8%, pH improvement from baseline of 80.1%.
- **Subject 3:** Satisfied; PPI Never; Regurgitation, None; GERD-HRQL score baseline/5-years = 30/6 (80% improvement); total time pH < 4 was 9.3%, pH improvement from baseline of 51.8%.
- **Subject 4:** Dissatisfied; PPI Seldom; Regurgitation, None: GERD-HRQL score baseline/5 years = 27/24 (11% improvement); total time pH < 4 was 6.4%, pH improvement from baseline of 93.2%.

To be noted, it is unlikely that subjects operated on due to GERD and pain would suddenly develop silent acid reflux. During the entire study, only one (*n* = 1) subject was both dissatisfied and had a pathologic total time with pH < 4 result, and only one subject in this study was viewed as a failure subject by the authors.

The other three out of four (*n* = 3/4) subjects never took PPIs, had no regurgitation, experienced 80–98% improvement in total GERD-HRQL score, had 52–80% improvement on 24-h pH testing results, and were satisfied. When providing a curve with normal values, 5% of the normal population will show values outside the defined normal range. In this study, these three subjects may be considered outliers within the normal population.

#### PPI use

The regular daily intake of PPI medication at various timepoints throughout the study period is presented in Table 4. At 5-year follow-up, 97.7% (*n* = 43/44) were not utilizing daily PPI therapy. The one (*n* = 1) subject using PPIs did not complete 24-h pH testing to objectively confirm indication of acid reflux, and this subject started with PPI therapy after falling ill with serious kidney disease after 4-year follow-up. With inclusion of the three (*n* = 3) COVID-affected subjects (i.e., two deaths and one bedbound with long-COVID) using available 3- (*n* = 2) and 4-year (*n* = 1) data from before illness with COVID-19, 97.9% (*n* = 46/47) of subjects were not utilizing daily PPI therapy. Additionally, none of the three subjects that terminated the study early took PPIs at 6-month (*n* = 2) and 3-month (*n* = 1) follow-up.

**Table 4:** PPI usage–Full analysis set

Daily, regular PPI intake	Months/n (%)						
	Baseline	6	12	24	36	48	60
**No**	**0 (0%)**	**47 (97.9%)**	**43 (93.5%)**	**44 (93.6%)**	**47 (100%)**	**44 (95.7%)**	**43 (97.7%)**
**Yes**, due to GERD with pathologic 24 h pH	**49 (98%)**	**0 (0%)**	**0 (0%)**	**1 (2.1%)**	**0 (0%)**	**0 (0%)**	**0 (0%)**
**Yes**, not due to acid reflux–normal 24 h pH	1 (2%)*	1 (2%)	2 (4.3%)	2 (4.2%)	0 (0%)	2 (4.3%)	0 (0%)
**Yes**, 24 h pH is missing	0 (%)	0 (0%)	1 (2.2)^†^	0 (0%)	0 (0%)	0 (0%)	1 (2.3)
**Total, n**	50	48	46	47	47	46	44**

^*^24-h pH total time 4.3% and esophagitis grade B during endoscopy

^**^Excluding 3 subjects lost to follow-up due to COVID-19, well-treated without PPI use at 3-year (*n* = 1) and 4-year (*n* = 2) follow-up (i.e., two deaths and one long-COVID)

^†^Normal pH at 5 years

*GERD* gastroesophageal reflux disease; *PPI* proton pump inhibitor

Bold text highlights key results described in the Results section

#### Regurgitation

**Fig. 4:**
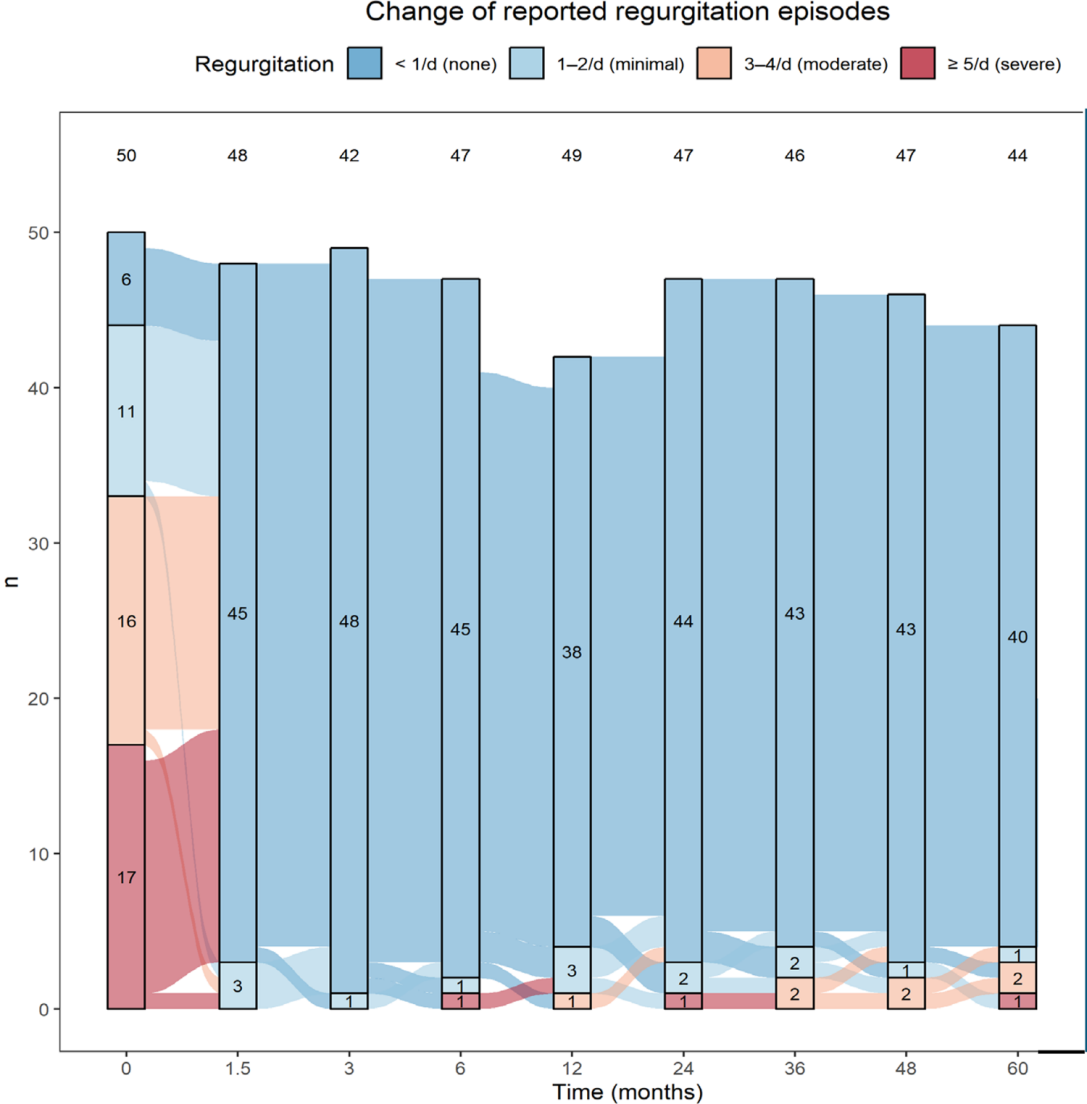
Change in regurgitation over time. The incidence of regurgitation immediately and substantially decreased which was sustained in the long-term for 5 years with 93.2% of subjects experiencing no or minimal regurgitation. The number within each differently colored bar represents the number of subjects with regurgitation episode frequencies of < 1/day (none), 1–2/day (minimal), 3–4/day (moderate), and ≥ 5/d (severe), respectively. The numbers at the top of each follow-up period represents the sample size of available regurgitation data

Figure 4 illustrates in detail the changes in reported regurgitation throughout the study period. Including the three COVID-affected subjects with available data at 3- and 4-year follow-up, 93.6% (*n* = 44/47) of subjects reported no or minimal regurgitation at the 5-year follow-up visit. This finding demonstrates the effectiveness of the device in treating acid reflux, as it successfully prevents regurgitation in most subjects over a long-term follow-up period.

## Safety outcomes

During the entire 5-year study period, no device-related serious or non-serious events (SADEs or ADES) occurred:

- No device deficiencies.
- No cases of migration.
- No cases of device explantation.
- No esophageal dilatations were performed.

### Dysphagia

During the entire study period between 2 weeks of surgical recovery and 5-year follow-up, 97.9% (*n* = 46/47) of subjects reported no AE dysphagia. One subject had mild dysphagia directly after surgery that lasted for 2 weeks, viewed as normal surgical recovery. No patient had unresolved AE dysphagia at 5 years. All food passageway-related AEs are presented in a separate article, including details of dysphagia [26], in which 97.9% of subjects reported no AE dysphagia or AE odynophagia, respectively, gas-bloating improved or was eliminated in 95.7% of subjects, and the ability to belch/vomit was completely (100%) maintained at 5 years.

### Re-herniation, device dislocation, or migration (evaluated by 5-year contrast-swallow X-ray)

The results of contrast-swallow x-ray imaging are presented in Table 5. This investigation was primarily performed at 6 months follow-up and repeated at the 5-year follow-up visit. No FAS subjects presented with re-herniation, device dislocation, or device migration. These findings suggest that the device maintains its intended position and effectiveness over a long-term period.

**Table 5:** Results of 5-year contrast-swallow x-ray–Full analysis set

Finding on contrast-swallow x-ray*	5-year Visit (*N* = 40), *n* (%)
Re-herniation	0 (0%)
Device dislocation	0 (0%)
Device migration	0 (0%)

^*^These results are unchanged since contrast-swallow x-ray was performed at 6 months

### ADEs, SADEs, SAEs, and non-serious AEs

As mentioned, no device-related adverse outcomes, ADEs or SADEs, occurred during the entire 5-year study period. A total of 16 procedure-related AEs occurred in 11 subjects during the study with 88% occurring before the 1-year visit. The most common AEs were unrelated to GERD and included gastritis (i.e., eight cases in *n* = 7 subjects), upper abdominal pain typical for gastritis (i.e., five events in *n* = 5 subjects), Coronavirus infection (i.e., five events in *n* = 5 subjects), and dyspepsia (i.e., four events in *n* = 4 subjects). Notably, no esophageal dilatations were performed.

#### Procedure-related SAEs

Five (*n* = 5) subjects experienced seven (7) procedure-related SAEs, presented in Table 6. Notably, all procedure-related SAEs occurred prior to the 1-year follow-up visit, of which all but one occurred in the first 30 days postoperatively, had a short duration, and were successfully resolved. Two (*n* = 2) subjects were reported as having severe SAEs. One (*n* = 1) subject was diagnosed and treated for bleeding from the short gastric vessels with surgical drainage of hematoma wherein the subject fully recovered (i.e., intraabdominal hemorrhage) and the bleeding had stopped spontaneously. One (*n* = 1) subject was diagnosed with infection with abdominal abscess, mediastinal abscess, and empyema (i.e., three events in one subject, although the same infection) 4 days after implantation. No damage or leakage was observed from the esophagus or stomach. These events were considered related to abdominal surgery in general, the infection did not affect the device in its enclosed pouch, and the subject recovered fully treated. The remaining three (3) SAEs were moderate or mild. One (n = 1) subject had part of a subcutaneously placed and broken surgical needle (i.e., foreign body) removed 1 day after device implantation. The subject completely recovered. One (*n* = 1) subject was diagnosed with loose plication sutures (i.e., suture rupture) that was completely resolved by re-suturing performed electively 8 months post-surgery. This was considered a learning curve experience of the procedure due to too much fat in the suture line. The most cranial plication suture had come loose and caused the fundus (with the implanted device intact) to move slightly away from the esophagus and downwards. After free dissection, the fundus was repositioned and re-sutured to the esophagus in the same way as the original procedure with a successful result (i.e., total GERD-HRQL score of 0 after the procedure). One (*n* = 1) subject was diagnosed with postoperative pleuritis (i.e., pleurisy). The subject recovered successfully. Thus, all procedure-related SAEs were resolved, and patients were well-treated.

**Table 6:** Procedure-related SAEs–Safety analysis set

Complication	Outcome	Time to Onset	Duration	Total (*n* = 50)		
				Mild	Moderate	Severe
				*n* (%)	*n* (%)	*n* (%)
Procedural complications				1 (2%)	1 (2%)	0 (0%)
Foreign body, broken needle subcutaneously	Recovered/resolved	0 to ≤ 30 days	1 day	1 (2%)	0 (0%)	0 (0%)
Suture rupture	Recovered/resolved	> 4.5 to ≤ 9 months	1 day	0 (0%)	1 (2%)	0 (0%)
Infections*				0 (0%)	0 (0%)	1 (2%)
Infection with abdominal and mediastinal abscess and empyema	Recovered/resolved	0 to ≤ 30 days	30 days	0 (0%)	0 (0%)	1 (2%)
Gastrointestinal disorders				0 (0%)	0 (0%)	1 (2%)
Intraabdominal hemorrhage	Recovered/resolved	0 to ≤ 30 days	1 day	0 (0%)	0 (0%)	1 (2%)
Respiratory, thoracic, and mediastinal disorders				0 (0%)	1 (2%)	0 (0%)
Pleurisy	Recovered/resolved	0 to ≤ 30 days	11 days	0 (0%)	1 (2%)	0 (0%)
Any SAE				1 (2%)	2 (4%)	2 (4%)

^*^One infection with three diagnoses–all occurred in one (*n* = 1) subject

*AE* adverse event; *n* number of subjects

#### Procedure-related AEs (non-SAEs)

Nine (9) procedure-related AEs (non-SAEs) occurred as shown in Tables 7 and 8. However, only two (2) procedure-related AEs were observed between 1- and 5-year follow-up: One (*n* = 1) case of moderate dyspepsia that remains ongoing and one (*n* = 1) case of temporary mild dysphagia with full recovery. The case of postoperative dysphagia was mild in severity and temporary with symptoms resolved by 5-year follow-up. This subject had GERD-HRQL dysphagia subscore of 5.0 at baseline compared to score 2.0 at the time of AE event.

**Table 7:** Procedure-related post-surgical AEs (non-SAEs)–Safety analysis set

Complication	Outcome	Time to Onset	Duration	Total (*n* = 50)		
				Mild	Moderate	Severe
				*n* (%)	*n* (%)	*n* (%)
Gastrointestinal disorders						
Dyspepsia	Not recovered	> 1.5 to ≤ 2.5 years	-	0 (0%)	1 (2%)	0 (0%)
Dysphagia*	Recovered/Resolved	> 2.5 to ≤ 3.5 years	3 years*	1 (2%)	0 (0%)	0 (0%)
Procedural postoperative complications**						
Procedural pneumothorax**	Recovered/Resolved	0 to ≤ 30 days	1 day	1 (2%)	0 (0%)	0 (0%)
Intestinal paralysis an extra day postoperative**	Recovered/Resolved	0 to ≤ 30 days	1 day	1 (2%)	0 (0%)	0 (0%)
Incisional hernia with abdominal pain**	Patient decided no surgery needed	> 4.5 to ≤ 9 months	-	0 (0%)	1 (2%)	0 (0%)
Any AE				3	2	0

*AE* adverse event; *n* number of subjects

^*^Subject with severe dysphagia preoperative with maximum GERD-HRQL score 5 on the dysphagia question, which was reduced to score 2 at AE event

^**^These adverse events are related to laparoscopic surgery in general and not specifically to the RefluxStop procedure

**Table 8:** Procedure-related AEs (non-SAEs) during surgery– Safety analysis set

Complication	Outcome	Time to Onset	Duration	Total (*n* = 50)		
				Mild	Moderate	Severe
				*n* (%)	*n* (%)	*n* (%)
Surgical procedure						
Hepatic lesion small*	Recovered/Resolved during the primary surgery	0 to ≤ 30 days	During surgery	1 (2%)	0 (0%)	0 (0%)
Hemorrhage small due to adhesiolysis handled during primary surgery**	Recovered/Resolved during the primary surgery	0 to ≤ 30 days	During surgery	1 (2%)	0 (0%)	0 (0%)
Any AE				2	0	0

*AE* adverse event; *n* number of subjects

^*^A small lesion on the liver due to the instrument holding the liver is common, need some blood coagulation, but would normally be viewed as less significant. This is not related to the RefluxStop procedure per se

^**^An adhesiolysis during primary surgery is not an AE, the surgeons always need to adapt to the situation inside the body and careful coagulation is needed when more adherences than normal are divided. Hemorrhage due to adhesiolysis during surgery is not related to the RefluxStop procedure per se

## Discussion

RefluxStop is designed to restore the correct physiologic anatomy in acid reflux sufferers. The device is placed in a pocket outside the fundic wall in a free-hanging pouch protruding into the stomach cavity. Treatment is achieved by reinstating the normal physiological anatomy. RefluxStop also maintains the restored anatomy. It treats acid reflux disease without encircling the food passageway and consequently, reducing side effects. This was not clear eight years ago, when we started performing this procedure, it was unclear how well RefluxStop would treat reflux symptoms when not affecting the food passageway. Today, we have a long experience of up to 8 years with RefluxStop and present both objective and patient-reported 5-year data as outlined herein, with the results showing that RefluxStop treats GERD effectively. If the procedure is performed as instructed, practically all patients should be well-treated for reflux symptoms. Furthermore, when using an implant, erosion/migration could always be a risk. Today, it can be concluded that zero (0%) erosion/migrations occurred in the study thus far, likely due to the special design of the procedure.

Long-term treatment of GERD is fraught with ineffectiveness and sequelae non grata that has resulted in substantial treatment gaps with the current edicts of management (i.e., standard of care). Thus, achieving satisfactory clinical efficacy with acceptable adverse effect rates is arguably difficult using conventional measures. For instance, PPI-based medical treatment is refractory in up to 40% of cases [8] and may result in a plethora of adverse effects in those requiring lifelong treatment, as is common in GERD. This includes cardiovascular events, chronic kidney disease, digestive cancer, and infectious/parasitic disease, leading to excess mortality [1, 27].

Moreover, the standard-of-care anti-reflux surgical option, Nissen fundoplication, has a well-known association with anathema outcomes like dysphagia [13]. This similarly applies to acid reflux treatment via MSA which may also result in postoperative dysphagia [28]. The MSA procedure aims to strike a balance between dysphagia of worse severity from using smaller diameter sizing of the augmentation device and sacrificed treatment effect using larger-sized versions [29]. Alternative anti-reflux procedures, such as endoscopic therapies, have questionable objective treatment effects and only modest results in relatively non-severe patient groups [30, 31] with many being withdrawn due to a lack of true effectiveness and/or complications [30]. Long-term outcomes of Nissen fundoplication, the standard of care, were reported in an extensive systematic literature review of 63 randomized clinical trials (RCTs), and include a rate of dysphagia of approximating 29% after 5 years [13]. This article provides a valuable platform for comparison to the standard of care used herein.

Out of available fundoplication techniques, Nissen fundoplication was used for comparison since it is considered the gold standard (i.e., standard of care) as per several clinical practice guidelines like the 2022 American College of Gastroenterology (ACG) recommendations [32]. RCTs and one systematic literature review with meta-analysis comparing Nissen and Toupet fundoplication indicated that although there are some differences in postoperative dysphagia during short-term follow-up, outcomes seem to approximate after 1 year with no statistically significant difference in the long-term [33–35]. For instance, the randomized study by Qin et al. with 383 cases followed for 5.6 years concluded that no symptom recurrence occurred following Nissen fundoplication whereas 18 patients experienced symptom recurrence following Toupet fundoplication [34]. Furthermore, the cure rate of esophagitis was superior in the Nissen group (88.4%) as compared to the Toupet (67.7%) group and the incidence of dysphagia between groups was comparable at 1-year postoperatively (i.e., 1.5% and 0.8%, respectively), supporting Nissen fundoplication’s position as the standard of care.

An appropriate methodology for establishing de facto treatment effect involves validation of patient-reported outcomes with objective testing. This study reports the first data of long-term (≥ 5 years) clinical outcomes using the novel RefluxStop procedure in the treatment of chronic GERD. Our report correlates subjective and objective outcomes for a comprehensive understanding of the long-term safety and effectiveness of RefluxStop surgery in a highly controlled and validated study. Valid 5-year results necessitate a high rate of follow-up, and in this study all efforts had been taken to track and report the outcomes of each subject, even those no longer part of the study at the 5-year visit (as outlined in the results section on *Missing Subjects*).

### Important clinical learnings and considerations

Five important learnings were gained from this study, detailed discussion of which is expounded upon in the text that follows:

#### No encirclement of the food passageway

Standard-of-care treatment encircles and applies pressure on the food passageway based on the assumption that the sphincter between the stomach and esophagus is weakened, which new experience with RefluxStop indicates is not entirely the case since patients are well-treated without encircling the food passageway. When investigating side effects of standard-of-care Nissen fundoplication, according to the previously mentioned systematic literature review [13], the food passageway-related AEs are substantial. These side effects are rather burdensome and anathema for patients contributing to overall morbidity. For instance, Humphries et al. reported that dissatisfaction following laparoscopic fundoplication was most often due to new-onset symptoms such as dysphagia and gas-bloating, despite improvement in GERD [36].

There are several surgeons who have attempted more limited fundoplication procedures, such as the BICORN procedure performed in Germany, which was also a topic of a dissertation [37]; however, existing results are not comparable to RefluxStop data. A key difference with RefluxStop surgery is its device-based mechanism of action where the implant interacts with the diaphragm to maintain sufficient distance between the hiatus and LES to act as an anatomical stabilizing agent [38], simultaneously repositioning the LES and reconstructing the flap valve. Pressure variations in the chest during respiration leak out through the hiatal opening and affect the LES even at larger distances from the diaphragm, and the device invaginated in its stomach pouch provides a large fundal package left and dorsal to the hiatus to ensure that the LES-diaphragm distance is maintained in interaction with the diaphragm.

In the RefluxStop procedure, the food passageway is, as mentioned, not encircled. This is the a priori reason behind the limited number of subjects that reported an AE of dysphagia, which is 14 times less likely as per the collated and published figures for the standard of care [13]. The data on food passageway-related side effects including gas-bloating, the inability to belch and/or vomit, odynophagia, and dysphagia are reported in a separate complementary article [26] due to the extensive amount of data generated from this CE-study for its use in a PMA submission to the US FDA, using 5-year data.

#### Stable patient outcomes in the long-term (over 5 years)

The outcomes reported in published literature for 6-month, 1-, 3-, and 4-year follow-up remained stable over 5 years after RefluxStop surgery, as demonstrated by PPI cessation, contrast-swallow x-ray imaging, objective 24-h pH monitoring results, and improvement in GERD-related quality-of-life scores. Like 6-month results, PPI therapy in this RefluxStop study was discontinued in all but one subject (2.1%) at 5 years. The actuality of only one patient using PPIs at 5-year follow-up highlights the sustained effectiveness of the device in managing GERD, which aligns well with the excellent pH results observed and demonstrates continued treatment success with decreased reliance on anti-reflux medication. Objective evaluation via contrast-swallow x-ray imaging showed no device dislocation, device migration, or re-herniation at 6 months. This imaging study was repeated at 5 years with no change: no device dislocation, device migration, or re-herniation. The acid exposure time on 24-h pH monitoring, an objective measure of acid reflux, was reduced from a mean value of 16.5% (percentage total time with pH < 4) at baseline to 0.82% at 6 months and was sustainedly low (1.57%) at 5 years. Ultimately, 24-h testing showed a mean improvement of 90.4% at 5-year follow-up compared to baseline. As shown in Fig. 3, the graphical presentation of 6-month and 5-year data is nearly identical, where the pathologic threshold that is often defined as > 4.5% of total time or based on research of more sensitive wireless Bravo capsules (used in this study) having values ≥ 5.9% shown to be pathologic [25]. No subject was dissatisfied with pathologic pH at 6 months, meanwhile at 5 years only one subject was dissatisfied and had a slightly pathologic pH (i.e., total time with pH < 4 was 6.4%).

At any time after surgery, n = 47/50 subjects underwent 24-h pH monitoring, and the graphical presentation of this data is quite similar to exclusively 5-year data (see Fig. 3a and c). For indirect comparison, the FDA trial for the MSA device showed 36% failure on 24-h pH testing as early as 1 year with the definition of ≥ 4.5% *or* < 50% improvement in acid exposure time (i.e., pH < 4) [24], compared to zero (0%) in the RefluxStop study. When failure is solely defined by acid exposure time ≥ 5.9% (for Bravo Capsules), two subjects failed 24-h pH-monitoring, however, one of these two is well-treated based on all other parameters including improvement of pH and questionnaire results.

Objective outcomes in the present study (e.g., pH testing) were reinforced by improvements in GERD-related quality-of-life scores after RefluxStop surgery. The total GERD-HRQL score (10 questions) decreased from a median of 29.5 at baseline to 2.0 at 6 months and 3.0 at 5 years, a 90.4% improvement at 5 years from baseline.

#### Patient safety-focused implant design and procedure technique

The design of the procedure and device invokes a unique surgical approach, employed to naturally and intentionally limit the potential for AEs. The RefluxStop device is placed on the outside of the stomach and encapsulated in a free-hanging pouch of fundic wall. Thus, any potential harm caused by the device is with high likelihood only possible if RefluxStop leaves the pouch. This pouch hangs in a tear-drop shape protruding into the stomach cavity and is closed by a row/line of sutures superiorly. As such, in the rare event that the device erodes or migrates (i.e., only occurring through bodily tissue), the implant enters in the stomach cavity as a result. No such event occurred in this study. Due to the design of the device in five segments, it first disintegrates into separate pieces as small as 15 mm that may easily pass through the digestive tract without incurring symptoms or re-operative measures. Thus, migration/erosion of the implant is presumably reduced in severity to an AE rather than an SAE, although that being said, no such migration/erosion event occurred in this study.

The safety of the RefluxStop procedure is further emphasized by the low rate of procedure-related AEs that occurred in this study:

- Two serious SAEs occurred that were directly related to the procedure: one case of bleeding from the short gastric vessels, with drainage of hematoma that spontaneously resolved; and one case of infection (with abscesses) that did not involve the device due to its complete and isolating encapsulation in the fundic pouch. Such SAEs are well-known and similarly applicable with the standard of care as well as alternative procedures. Both subjects recovered completely and were well-treated.
- No device-related AEs or SAEs occurred during the entire study period.
- Importantly, no cases of device explant, device migration/erosion, or esophageal dilatation occurred during the entire study period, reflecting the robust safety profile of the device.
- Outcomes of food passageway-related AEs such as dysphagia, inability to belch/vomit, and gas-bloating are infrequent or non-existent, presented in a separate complementary article [26] where 97.9% of subjects reported no AE dysphagia or AE odynophagia (both subdomains of the GERD-HRQL questionnaire), respectively, 95.7% of subjects reported improved or eliminated gas-bloating, and all (100%) subjects reported a preserved ability to belch/vomit at 5 years. Indirect analysis to the standard of care can be made by meta-analyses and literature reviews of high quality.
- The extensive literature review on Nissen fundoplication previously mentioned [13], which included 63 RCTs, demonstrated high rates of dysphagia (28.9%), odynophagia (16%), gas-bloating (52.7%), and inability to belch/vomit (39.8%) at 5 years that are substantially elevated in relation to RefluxStop results in this pivotal study.

The favorable safety profile of RefluxStop surgery is further supported by only two minor procedure-related AEs and no SAEs occurring between 1 and 5 years of this study: one case of temporary, resolved mild dysphagia and one case of moderate dyspepsia. Overall, this low rate of AEs is most likely attributable to the design of the RefluxStop implant, loose fundus invagination as a standardized surgical step, and flexible/dynamic physiological interaction that avoids encircling or exerting pressure on the food passageway, as outlined above.

#### Comparison (Naïve) with published outcomes of existing methods

Although head-to-head study with other anti-reflux procedures is currently lacking, indirect comparison of the long-term data from this study demonstrates notable improvement in clinical outcomes when reviewing the literature on standard-of-care Nissen fundoplication and MSA.

For instance, the adverse outcomes associated with standard-of-care Nissen fundoplication appear to occur more frequently, as shown by the recently published literature review on Nissen fundoplication [13]. In this publication, gas-bloating occurred in 52.7% of patients and AE dysphagia occurred in approximately 29% of Nissen fundoplication cases at 5 years, whereas the dysphagia AE rate was 2% after RefluxStop at 5-year follow-up [26]. RefluxStop results are highly dissimilar to conventional surgery by indirectly comparing to this literature review, and all food passageway-related AEs are detailed in a separate complementary report [26]. MSA generally impacts the lower esophagus to the degree that 68% of patients may experience postoperative dysphagia at 1 year [12]. The combination of explantation and esophageal dilatation occurs in 8.3% of patients up to 1 year after MSA as per one study [39], however, there are many examples of higher rates of esophageal dilatation (17–31%) after MSA alone [29, 40]. MSA explantation may occur in 5.5% of patients at an average of 29 months, as per a specialist anti-reflux center [41].

This disparity and seeming benefit in favor of RefluxStop is likely the result of the procedure and device design which intentionally circumvents esophageal encirclement or compression to reduce food passageway-related sequelae. The dissimilarity occurs in most parameters, to a varying degree: PPI usage is 12% after Nissen fundoplication compared to 2% in this study; and the inability to belch and/or vomit occurred in 39.8% after Nissen fundoplication compared to 0% in this study [13]. Direct comparative study is currently underway to verify this assertion.

In terms of objective 24-h pH testing, Nissen fundoplication at the interval between 6 months to 1 year results in a mean acid exposure time of 3.3% [13] compared to 0.8% at 6 months in this trial. According to the FDA Memorandum for the MSA device, 36% of patients failed 24-h pH testing [24] compared to RefluxStop with zero (0%) failures using the same definition as in the Memorandum (wherein > 50% improvement of total time pH < 4 is also viewed as normal 24-h pH). Although these comparisons are naïve and indirect, the differences are quite substantial and repeated over the various parameters, which may indicate a de facto superiority to be further supported by head-to-head evaluation.

#### Post-market real-world data of the RefluxStop procedure

Overall, results from the available real-world data (i.e., several articles published or currently in peer review) further validate and reinforce the outcomes of this multi-publication pivotal CE trial [19–21], encompassing additional RefluxStop patients with data from several leading RefluxStop surgeons in centers across Germany, Switzerland, Austria, and the United Kingdom (UK). This data include complex patient demographics that may typically preclude optimal outcomes with other anti-reflux procedures, however, RefluxStop surgery has essentially shown consistency in safety and effectiveness regardless of complicating disease comorbidities.

Published reports of real-world data dedicated to IEM [23, 42, 43] and large hiatal hernia [22, 44] patients, as well as other studies with large hernia subgroups [45, 46], are such examples and corroborate the results of this study. Importantly, re-herniation is a common outcome following standard-of-care anti-reflux surgery (regardless of mesh use) that occurs in up to 55% of cases at 5 years in subjects with large hernia, according to a recent RCT [47]. No such case of hernia recurrence was visible at the 5-year contrast-swallow x-ray in the present study, although performed in a group with no hiatal hernias > 3 cm in size at baseline.

As an example, patients with chronic GERD (*n* = 79) underwent the RefluxStop procedure in a German hospital with a mean (SD) follow-up near 1 year (11 [4.4] months) [46]. Median (IQR) improvement in total GERD-HRQL score of 100% (90.2–100%) from real-world settings compared similarly to 95.1% and 90% at 1 and 5 years, respectively, in this pivotal study. Significant reduction in PPI use was observed from a baseline of 94.9% to 2.5% at follow-up, compared to 2.1% in the pivotal study (both at 6 months and 5 years). All cases of preoperative dysphagia (7.6%) completely resolved. New-onset, mild dysphagia occurred in one subject (1.3%) which was also comparable with the pivotal study, where details are reported in the previously mentioned complementary article [26].

The consistency of clinical outcomes with RefluxStop surgery in both stricter and real-world settings demonstrates the robustness of RefluxStop’s design rationale and approach to the treatment of acid reflux.

### The impact on GERD-HRQL scores from other ailments

Today, GERD-HRQL has established itself as the standard for patient-reported outcomes in reflux patients managed by anti-reflux surgery. In our study, total GERD-HRQL scores demonstrated a median 90% improvement from baseline. However, as our study shows, the GERD-HRQL score has one notable weakness, namely that diseases other than acid reflux may affect questionnaire results. This strongly suggests that patient-reported quality-of-life scores should be supported with objective pH measurement needed to diagnose other such diseases. By far, the most common ailment that influences GERD-HRQL scores is gastritis, with a prevalence approximating 25–35% [48]. In this study, 12 subjects had symptoms of gastritis, of which eight were diagnosed with the disease. Therefore, to objectively confirm results it is important to support patient-reported outcomes by incorporating objective measures such as 24-h pH monitoring to provide additional clinical context, especially since RefluxStop does not treat gastritis (only GERD).

### Limitations and strengths

The main limitation of our study is the lack of a control group. However, the current published literature from real-world clinical experience is growing rapidly, providing a basis for broader safety and effectiveness outcome comparisons. Additional considerations for the generalizability of results are the sample size (*n* = 50) and the experience level or expertise of operating surgeons. Moreover, variability in complex patient characteristics such as hernia size, BMI, and atypical symptoms also limit generalizability of results. In real-world practice, there are patients with complex profiles, such as those with large hiatal hernia (> 3 cm), high BMI (> 35 kg/m^2^), previous unsuccessful anti-reflux surgery, and atypical GERD symptoms, in whom the generalizability of these results may be limited. Many such treatment areas such as large hernia and dysmotility have already been evaluated in real-world studies [22, 23, 42–46, 49, 50].

Due to the use of this prospective study for both CE and US FDA PMA (5-year data) purposes, this study is of high quality and includes an extensive data package, having been rigorously controlled and providing a broad statistical analysis with a high rate of follow-up. The clinical follow-up at 5 years was 92% when excluding subjects that succumbed to COVID-19. Missing subjects’ data from previous visits were presented.

Additionally, objective measures were performed at 5 years, which included both 24-h pH testing and contrast-swallow x-ray, in more than 90% of subjects remaining in the study. Contrast-swallow x-ray imaging was performed at 5 years to discern whether device-related safety signals (i.e., migration, dislocation, re-herniation, and penetration) occurred or identify any potential cause for diminished treatment effect (such as inadequate positioning of the device), providing additional clinical context. Both PPI usage and contrast-swallow imaging results paralleled expectation from comparison to objective 24-h pH monitoring, GERD-HRQL scores, and treatment overall. Investigator-initiated studies representative of real-world settings support these data. Although quality of life should always be an important consideration in therapy decision-making, better approximation of the true effectiveness of an intervention requires objective validation. As such, this study has shown favorable and sustained (i.e., long-term) results with RefluxStop surgery that are reflected in both objective evaluation and the patient experience.

## Conclusion

This study presents both patient-reported outcomes and objectively validated safety and effectiveness of the RefluxStop procedure for the treatment of GERD in the long-term (i.e., ≥ 5 years). Most importantly, the previously published, excellent results from 6-month and 1-, 3-, and 4-year follow-up were maintained and shown to be stable over the 5-year study period. The 5-year results provide strong indication that the RefluxStop procedure can offer sustained safe and effective relief from GERD symptoms and significantly reduce the need for ongoing medical therapy in the long-term with only one subject taking PPI medications at 5 years.

The median total GERD-HRQL score (i.e., 90% improvement from baseline) was validated by objective 24-h pH testing results, which improved by over 90%, and contrast-swallow x-ray imaging, which showed stable device position without any dislocation, migration, or re-herniation at 5 years. No cases of device-related AEs, migration, explant, device deficiency, or esophageal dilatation occurred during the entire 5-year study period, in line with published real-world outcomes from current clinical experience of similar patient populations. All procedure-related SAEs resolved, and subjects were well-treated.

Also, since the difference in indirectly compared outcomes in favor of RefluxStop is both significant and vast, via presently published literature reviews on the standard of care, this report provides an indication that there is a substantial opportunity for improvements on standard-of-care anti-reflux surgery with the novel RefluxStop procedure, to potentially further advance the GERD surgical treatment field moving forward.
